# Obituary

**Published:** 1902-02-15

**Authors:** 


### OBITUARY.

### DR. CHARLES J. ESSIG.

Dr. Charles J. Essig, the author of the well-known text-book on dental metallurgy and the editor of the "American Text-Book of Prosthetic Dentistry," died at his home in Pennsylvania, December 2, 1901. He has had a creditable professional career. Much of his life was spent in teaching, and he only resigned the chair of prosthetic dentistry in the University of Pennsylvania a few months before he died. He was an educated man with artistic feelings, and gave unselfishly of his time and talents to the profession and particularly to those preparing to enter it. He was sixty-one years old when he died.

